# Dietary Grape Seed Proanthocyanidin Alleviates the Liver Injury Induced by Long-Term High-Fat Diets in Sprague Dawley Rats

**DOI:** 10.3389/fvets.2022.959906

**Published:** 2022-08-03

**Authors:** Hao Yang Sun, Ai Xin Gu, Bi Ying Huang, Tong Zhang, Jian Ping Li, An Shan Shan

**Affiliations:** College of Animal Science and Technology, Institute of Animal Nutrition, Northeast Agricultural University, Harbin, China

**Keywords:** liver injury, grape seed proanthocyanidin, Wnt3a/β-catenin signaling pathway, endoplasmic reticulum stress, microRNA, lipid metabolism

## Abstract

In mammals, the liver is the most important organ that plays a vital function in lipid metabolism. Grape seed proanthocyanidin (GSPE) is a kind of natural polyphenolic compound primarily obtained from grape skin and seeds. Recent research found it had high bioavailability in defending against obesity, hyperlipidemia, inflammatory, oxidative stress, and targeting liver tissue. However, the mechanism of GSPE in regulating obesity induced by dietary high-fat (HF) was not fully understood, particularly the influences on liver functions. Therefore, this study aimed to investigate the effects of GSPE supplementation on the liver function and lipid metabolic parameters in rats fed HF diets long-term. A total of 40 healthy female Sprague Dawley rats were selected. After 8 weeks of obesity model feeding, the rats were randomly divided into four treatments: NC, standard diet; NC + GSPE, standard diet + 500 mg/kg body weight GSPE; HF, high-fat diet; HG + GSPE, high fat diet + 500 mg/kg body weight GSPE. Results indicated that long-term HF feeding caused severe liver problems including megalohepatia, steatosis, inflammation, and hepatocyte apoptosis. The supplementation of GSPE alleviated these symptoms. The results of the current experiment confirmed that GSPE addition up-regulated the expression of the Wnt3a/β-catenin signaling pathway, thereby restraining the liver cell endoplasmic reticulum stress and hepatocyte apoptosis. Furthermore, the microRNA-103 may play a role in this signal-regulated pathway. In summary, GSPE had a protective effect on the liver and the current experiment provided a reference for the application of GSPE in animal nutrition as a kind of natural feed additive.

## Introduction

In the last few decades, non-alcoholic fatty liver disease (NAFLD), a chronic liver disease, has become a global public health problem that impacts about a quarter of adults worldwide (1). As it develops, a series of pathological changes occur in liver tissues and cells such as high lipid accumulation, abnormal triglyceride cycle, inflammation, mitochondrial dysfunction, cell apoptosis, and endoplasmic reticulum stress (ERS) (2). It not only increases greatly the probability of liver-related diseases, but also causes extrahepatic medical conditions such as type 2 diabetes, cardiovascular, and cerebrovascular diseases (3). Although the exact pathogenesis of NAFLD is still not fully understood, it is associated with some metabolic abnormalities including lipid metabolism.

Grape seed proanthocyanidin (GSPE), a natural polyphenolic compound enriched in phenolic hydroxyl groups, is primarily found in grape skin and grape seeds. Its role in defending organs and tissues against obesity, hyperlipidemia, inflammatory, and oxidative stress is widely acknowledged (4). Margalef et al. (5) suggested the bioavailability of GSPE in targeting liver tissue; with dietary supplementation of 375 mg/kg of GSPE, the rats' liver had comparatively higher concentrations of GSPE flavonoid metabolites including catechin, epicatechin, and glucuronidation of methyl. Moreover, it was considered that microRNAs (miRNAs) may be a key factor in altering lipid metabolism in NAFLD (6). *In vivo* study confirmed that polyphenols treatment repressed fatty liver disease in hyperlipidemic mice, and the positive effects were relevant to the expression of lipid metabolism-related miRNAs (7). A recent study demonstrated that taking orally GSPE decreases the size of the liver and alleviates the level of steatosis in patients with NAFLD (8).

The endoplasmic reticulum, an essential site for secretory protein and lipid synthesis has a notable capacity to maintain steady lipid metabolisms in the liver and plasma. But, excessive accumulation of triglycerides in non-adipocytes can lead to ERS and activate stress signaling pathways thereby resulting in the dysregulation of lipid metabolism (9). ERS has been considered to play an important role in the initiation of NAFLD progression (10). Moreover, ERS can intervene in the functional expression of the proapoptotic BCL2-family members, which may eventually induce hepatocyte apoptosis (11). It was reported that NAFLD correlates with the severity of hepatic cell apoptosis (12). Hence, anti-apoptotic therapy may be one of the therapeutic methods worth trying. On the other hand, regulations on the lipid metabolism pathway are also considered to be an alternative therapeutic solution. Wnt is a secretory protein and plays an important role in cellular development and proliferation (13). Recently, researchers found that the Wnt family and signaling also had a relevant effect on lipid metabolism (14). For instance, it was found that Wnt signaling led to repression of liver steatosis and triglyceride concentrations in zebrafish, thereby regulating lipid metabolism (15). In European NAFLD cases, gene expression analysis showed that Wnt signaling pathways may be pivotal in NAFLD pathogenesis (16).

A high-fat diet causes obesity and fatty liver is one of the possible factors leading to NAFLD. How exactly GSPE alleviates the liver affected by HFD-induced obesity has not yet been studied. Our study, therefore, hypothesized that the inclusion of GSPE in high-fat diets may reverse liver injury by alleviating the cell apoptosis from ERS and restraining lipometabolism. This experiment was carried out to investigate the effects of dietary GSPE on the liver function and lipid metabolic parameters of rats fed with or without high-fat diets. It is anticipated that the data obtained from this study will provide basic support for the functional uses of GSPE.

## Materials and Methods

Female Sprague Dawley (SD) rats were purchased from Beijing Vital River Laboratory Animal Technology Co., Ltd (Beijing, China). Grape seed proanthocyanidin (GSPE) was purchased from Tianjin Jianfeng Natural Product R&D CO., Ltd. (Tianjin, China). GSPE in this study contained >95% proanthocyanidins as per the manufacturer's instructions. The protocols were performed in accordance with the guidelines for the care and use of laboratory animals approved by the Institutional Animal Care and Use Committee of Northeast Agricultural University.

### Animals and Feeding

A total of 40 healthy female SD rats about five to seven-week-old were individually housed under a standard room (21± 4°C) with a 12 h light/dark cycle. All rats consumed food and water *ad libitum*. After 1 week of acclimation, rats were randomly distributed into two treatments (n=20): (1) one group fed with a standard diet (12.95% kcal from fat, 24.02% kcal from proteins, and 63.03% kcal from carbohydrates) supplied by Beijing Keao Xieli Feed CO., Ltd. (Beijing, China); (2) another group fed with high-fat diet (52.64% kcal from fat, 17.07% kcal from proteins, and 30.29% kcal from carbohydrates). After 8 weeks of feeding, an obesity model was set up based on the data shared in our previous study (17). The standard diet group and high-fat diet group rats were further divided randomly into 2 groups respectively (n=10): including (1) the NC (standard diet), (2) the GSPE (GSPE based on standard diet), (3) the HF (high-fat diet), and (4) the HF+GSPE (GSPE based on high-fat diet) treatments. The GSPE group and the HF+GSPE group were given daily gavage of 500 mg/kg body weight GSPE by gavage administration. The GSPE was dissolved in saline. The body weight and feed intake of rats were weighed weekly. These treatments lasted for 4 weeks. The rats were anesthetized after 12 h fasting. Liver tissues were removed swiftly and weighed. The collected tissues were frozen at −80 °C until analysis.

### Histopathology

The liver sample was fixed with a 10% neutral-buffered formalin solution for at least 24 h. The fixed specimens were dehydrated through a series of ethanol, cleared in xylene, and embedded in paraffin. The liver sections were cut into 4-μm sections and rehydrated through a series of incubations in xylene and ethanol solutions and then stained with hematoxylin and eosin (H&E). Sections were analyzed under light microscopy.

### Wnt3a/β-Catenin Pathway

Wnt3a and β-catenin levels in renal tissues were assessed using Rat Protein Wnt3a ELISA Kit (mlbio, Shang Hai, China) and Rat beta-catenin ELISA Kit (mlbio, Shang Hai, China), respectively. The manufacturer's instructions were followed exactly.

### Quantitative Real-Time PCR

TRIzol reagent was used to isolate Total RNA including miRNA from liver tissues, and RNA samples were purified and frozen at −80 °C until assay. cDNA was synthesized by reverse transcription of 5 μl total RNA using Prime Script RT reagent Kit, with gDNA Eraser according to the manufacturer's protocol as described. Primer sequences of target genes were designed from published GenBank and synthesized by Sangon (Shanghai, China) (Table 1). In this study, β-actin was used to normalize the expression of target gene transcripts. The sample was centrifuged momentarily and carried out on the Applied biosystem 7500 Real-Time PCR thermal cycler apparatus at the matched program (40 cycles of 95 °C for 5 s, 60 °C for 34 s). All PCR reactions were performed in triplicate and mRNA was evaluated by the 2-ΔΔct method as described previously.

**Table 1:** Primer sets for qPCR.

**Gene**	**Forward primer (5^**′**^-3^**′**^)**	**Reverse primer (5^**′**^-3^**′**^)**
miR-103	ACACTCCAGCTGGGGGCTTCTTTACAGTGCTG	TGTCGTGGAGTCGGCAATTC
PPARγ	GCCCTTTGGTGACTTTATGGAG	GCAGCAGGTTGTCTTGGATGT
FAS	ACCTCATCACTAGAAGCCACCAG	GTGGTACTTGGCCTTGGGTTTA
ATF6	GGATTTGATGCCTTGGGAGTCAGAC	ATTTTTTTCTTTGGAGTCAGTCCAT
CHOP	CCTTCACTACTCTTGACCCTG	CACCACTCTGTTTCCGTTTC
Bax	GAGGATGATTGCTGATGTG	AGTTGAAGTTGCCGTCTG
Bcl-2	GTGGCCTTCTTTGAGTTCGGT	CATCCCAGCCTCCGTTATCC
TNF-α	GATCGGTCCCAACAAGGAGG	GTGAGGAGCACATAGTCGGG
IL-6	AGCGATGATGCACTGTCAGA	TAGCACACTAGGTTTGCCGA
IL-1β	GACTTCA CC ATGGAACCCGT	GGAGACTGCCCATTCTCGAC
β-actin	GCAGAAGGAGATTACTGCCCT	GCTGATCCACATCTGCTGGAA

### Western Blot Analysis

The liver tissues were stored at −80°C. Tissue samples were homogenized in ice-cold RIPA lysis buffer (Millipore, Billerica, MA, USA) for protein extraction. Tissue debris was removed by centrifugation, and the resulting supernatants were collected and analyzed for protein concentration by the BCA protein assay kit. The protein was separated on a 10% SDS polyacrylamide gel and then transferred to nitrocellulose membranes (Beyotime Biotech, Shanghai, China). The membranes were incubated with specific primary antibodies overnight at 4°C. The primary antibodies included anti-mouse β-actin (Beyotime Biotech, Shanghai, China), anti-rabbit ATF6, anti-rabbit CHOP, anti-rabbit Wnt3a, and anti-rabbit β-catenin (Company ABclonal, Inc., Wu Han, China). After washing, the membranes were allowed to react with diluted horseradish peroxidase-conjugated secondary antibodies, including goat anti-rabbit IgG antibody and goat anti-mouse IgG (Beyotime Biotech, Shanghai, China) at room temperature for 2 h. An enhanced chemiluminescence system (Beyotime Biotech, Shanghai, China) was used to visualize antibody-antigen complexes.

### Statistical Analysis

All data were analyzed based on a 2 × 2 factorial arrangement using the general linear model procedure of the SAS software (SAS Inst. Inc., Cary, NC). The individual rats were considered as the experimental unit. The statistical model included the main effects of the GSPE and dietary fat, as well as the interaction between GSPE and dietary fat. Means were separated using Tukey's Test. GraphPad Prism 5.0 software was used for data plotting. Variability in the data was expressed as the pooled standard error of the means (SEM). A *P* < 0.05 was considered to denote statistical significance.

## Results

### Effects of GSPE on Liver Performance in Rats Fed Long-Term High-Fat Diet

**Figure 1:**
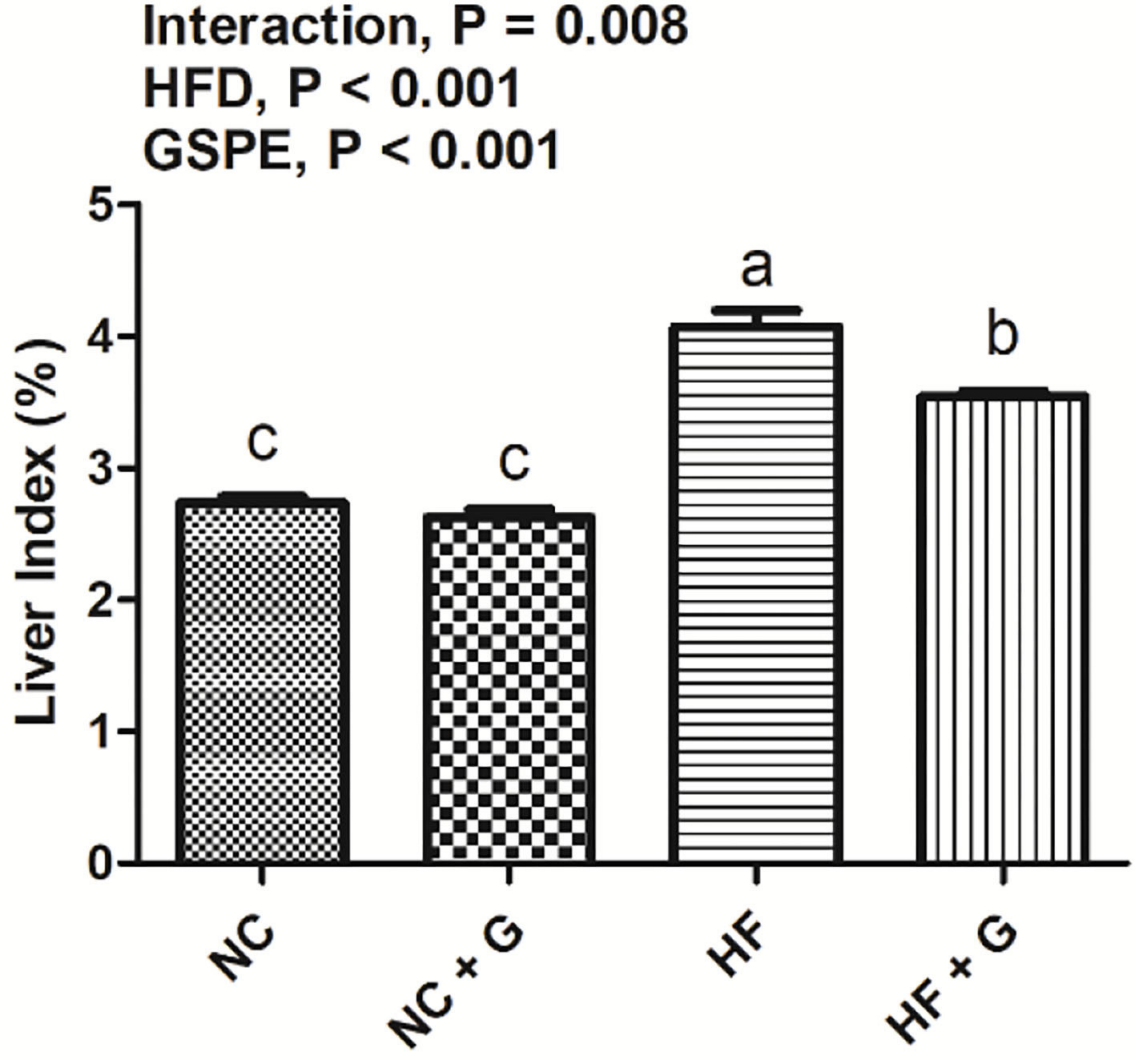
Effects of GSPE on liver index in rats fed long-term high-fat diet. Data were expressed as the mean ± SEM. Different letters means with different superscripts differ (*P* < 0.05). NC, standard diet; NC + G, standard diet + 500 mg/kg body weight GSPE; HF, high fat diet; HF + G, high fat diet + 500 mg/kg body weight GSPE.

**Figure 2:**
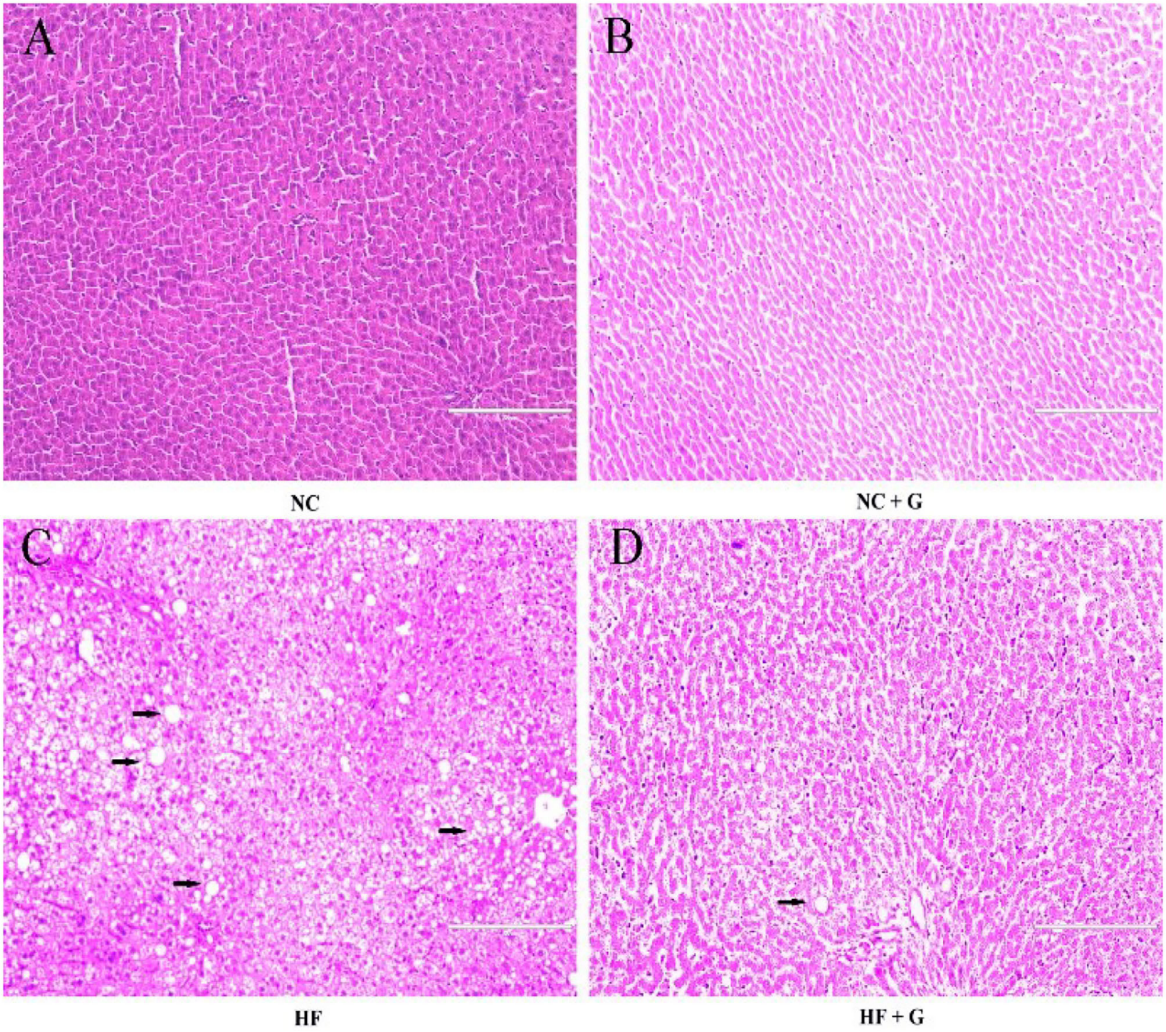
**(A)** NC, standard diet; **(B)** NC + G, standard diet + 500 mg/kg body weight GSPE; **(C)** HF, high fat diet; **(D)** HF + G, high fat diet + 500 mg/kg body weight GSPE.

In this experiment, the liver indexes (the ratio of liver weight and body weight) and cell morphological sections were considered as the liver performance. As shown in Figure 1, all rats fed with HF diets had higher (*p* < 0.05) liver indexes than those fed with NC diets. Compared with these non-supplementation treatments, the addition of GSPE observed lower liver indexes. Significant interactions were noted between dietary fat level and GSPE supplementation (*p* < 0.05). Rats in the HF group had the highest (*p* < 0.05) liver index than those in the other three groups. Liver index in HF + GSPE was also significantly (*p* < 0.05) higher than NC and NC + GSPE treatments. The liver morphological sections are shown in Figure 2. Compared with NC groups, hepatocytes in rats from HF diets observed adipose and injury. Furthermore, the most serious ones occurred in the HF treatment group, which showed circular lipid droplets filled in the cytoplasm, some of the cells were vacuolated and the nucleus had disappeared, and whole cells were filled with fat along with inflammatory infiltration. Protective responses were observed in the hepatocytes from HF + GSPE-fed rats. The fatty degeneration was alleviated, part cell morphology returned, the lipid droplets reduced or disappeared, and no inflammatory cells were observed.

### Effects of GSPE on Lipid Metabolism Genes' Relative Expression in the Liver of Rats Fed Long-Term High-Fat Diet

**Figure 3:**
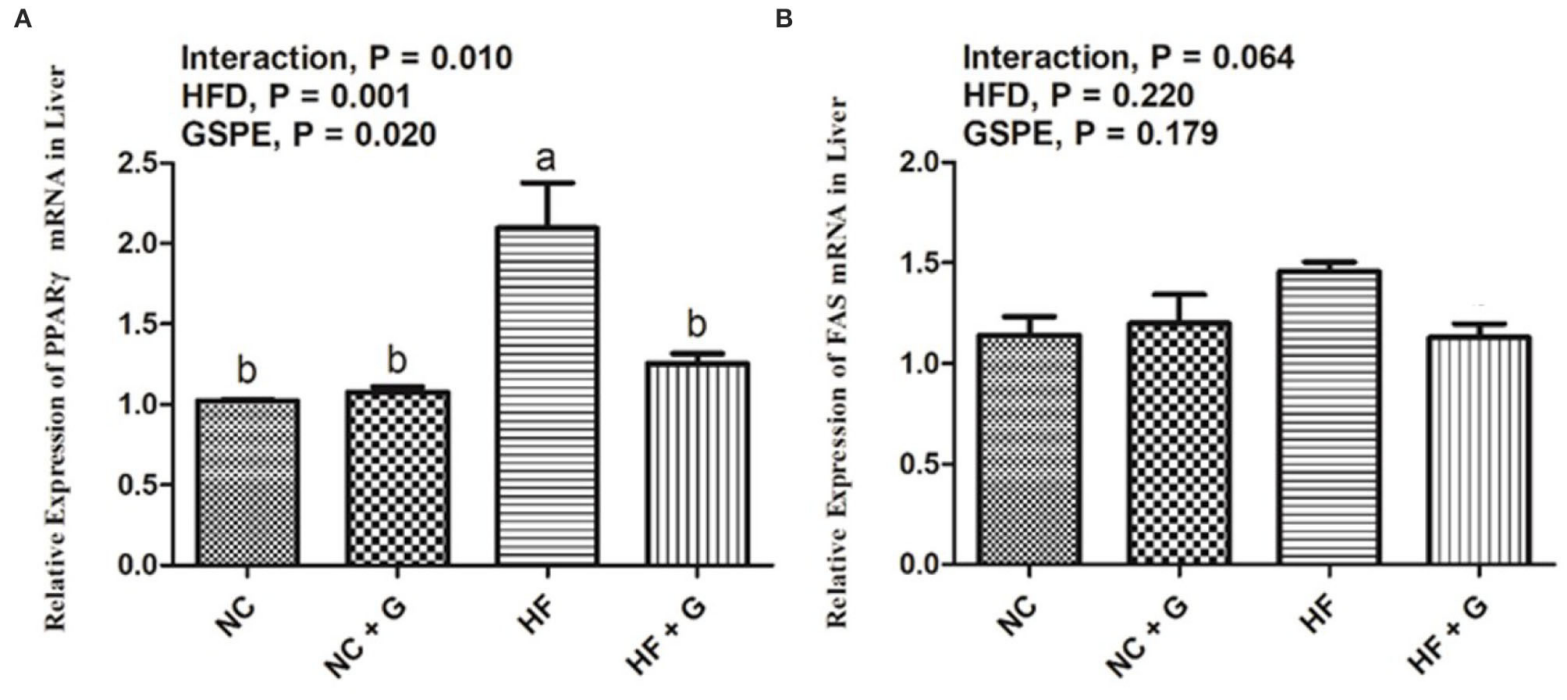
Effects of GSPE on relative expression of lipid metabolism genes of liver in rats fed long-term high-fat diet [**(A)** PPARγ mRNA expression; **(B)** FAS mRNA expression]. Data were expressed as the mean ± SEM. Different letters means with different superscripts differ (*P* < 0.05). NC, standard diet; NC + G, standard diet + 500 mg/kg body weight GSPE; HF, high fat diet; HF + G, high fat diet + 500 mg/kg body weight GSPE.

Peroxisome proliferators activate the receptor γ (PPARγ) and fatty acid synthetase (FAS) are two fat synthesis-related genes. As shown in Figure 3A, rats in HF groups had higher (*p* < 0.05) expression on mRNA of PPARγ compared with the rats in NC groups, but no effects on FAS expression (Figure 3B). Also, significantly (*p* < 0.05) higher PPARγ mRNA expression was observed in the GSPE supplementation treatment group than that in these non-addition treatment groups. Moreover, significant interactions were shown between dietary fat and GSPE for the PPARγ mRNA expressions. HF group had the highest (*p* < 0.05) expression among the treatments. However, there were no significant influences on FAS mRNA expression among the treatments.

### Effects of GSPE on the Expression of Endoplasmic Reticulum Stress mRNA and Proteins in the Liver of Rats Fed Long-Term High-Fat Diet

**Figure 4:**
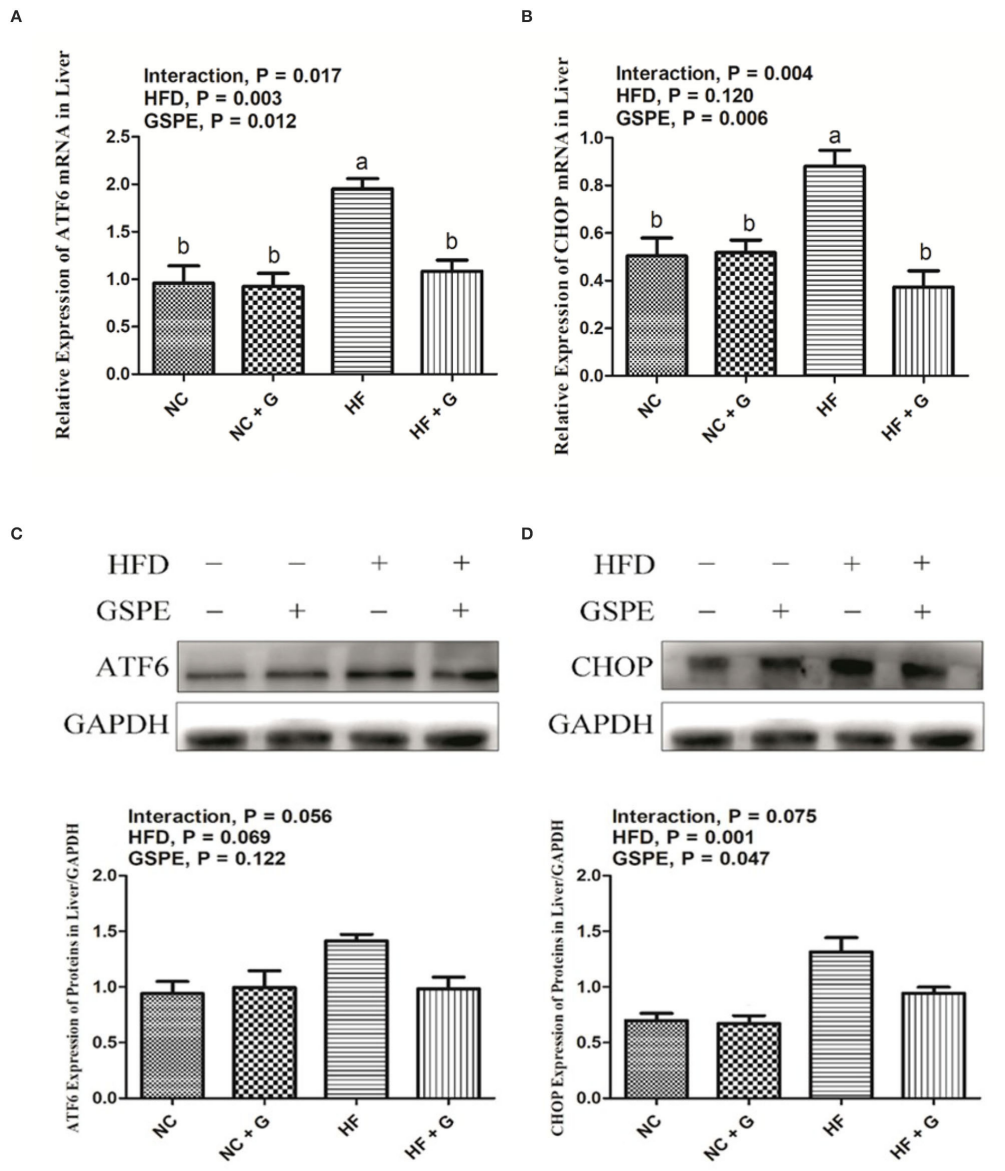
Effects of GSPE on relative expression of endoplasmic reticulum stress of liver in rats fed long-term high-fat diet [**(A)** ATF6 mRNA expression; **(B)** CHOP mRNA expression; **(C)** Protein production on ATF6 gene; **(D)** Protein production on CHOP gene]. Data were expressed as the mean ± SEM. Different letters means with different superscripts differ (*P* < 0.05). NC, standard diet; NC + G, standard diet + 500 mg/kg body weight GSPE; HF, high fat diet; HF + G, high fat diet + 500 mg/kg body weight GSPE.

Activating transcription factor 6 (ATF6) and CCAAT/enhancer-binding protein homologous protein (CHOP) are two of the main factors during the ERS signal pathway. As described in Figures 4A,B, higher dietary fat resulted in higher (*p* < 0.05) expressions on mRNA expression of ATF6; whereas, it did not affect the mRNA expression of CHOP compared with the samples from normal diets. Supplementation of GSPE observed significant decreases in both the expression of ATF6 and CHOP mRNA than those no-supplement ones. Interactions between GSPE and dietary fat were observed in the expressions of both ATF6 and CHOP. These mRNA expressions were all reduced in the HF + GSPE treatment than in the other treatments. The results of protein expressions on ATF6 and CHOP genes are shown in Figures 4C,D. For the protein expression on ATF6, there were no effects among treatments on dietary fat, GSPE, or the interaction. Higher dietary fat had significantly (*p* < 0.05) higher protein expression on CHOP than those fed with normal diets. Also, dietary inclusion of GSPE significantly (*p* < 0.05) decreased the CHOP protein expression compared with those clean ones. There were no interaction effects on CHOP protein expression between dietary fat and GSPE supplementation.

### Effects of GSPE on the Relative Expression of Apoptosis mRNA in the Liver of Rats Fed Long-Term High-Fat Diet

**Figure 5:**
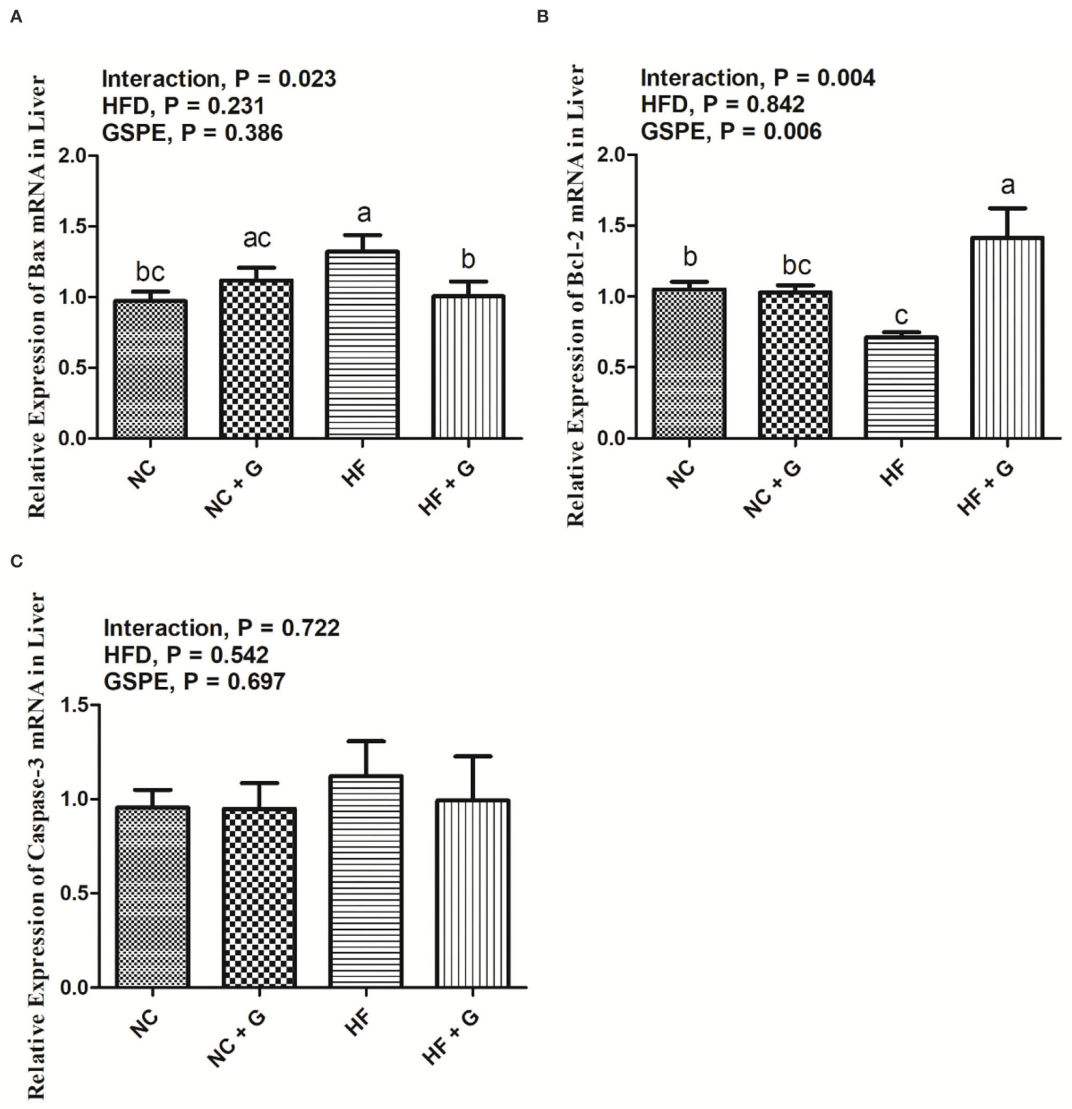
Effects of GSPE on relative expression of apoptosis proteins of liver in rats fed long-term high-fat diet [**(A)** Bax mRNA expression; **(B)** Bcl-2 mRNA expression; **(C)** Caspase-3 mRNA expression]. Data were expressed as the mean ± SEM. Different letters means with different superscripts differ (*P* < 0.05). NC, standard diet; NC + G, standard diet + 500 mg/kg body weight GSPE; HF, high fat diet; HF + G, high fat diet + 500 mg/kg body weight GSPE.

In the current experiment, the genes of Bax, Bcl-2, and Caspase-3 were considered as the genes that regulate apoptosis. As shown in Figure 5, no significant effects were observed on the expression of the three mRNA in HF treatments compared with those fed normal diets. Similarly, GSPE supplementation also did not influence the mRNA expression of Bax and Caspase-3. However, the inclusion of GSPE greatly (*p* < 0.05) improved the Bcl-2 mRNA expression in rats' livers. Interactions were noted between dietary fat and GSPE for Bax and Bcl-2 mRNA expressions. The Bax mRNA in HF + GSPE group showed lower (*p* < 0.05) expression than that in NC + GSPE and HG treatments; moreover, the addition of GSPE in HF treatment observed the highest (*p* < 0.05) expression of Bcl-2 mRNA compared with the other three treatments.

### Effects of GSPE on the Relative Expression of Inflammatory Cytokines mRNA in the Liver of Rats Fed Long-Term High-Fat Diet

**Figure 6:**
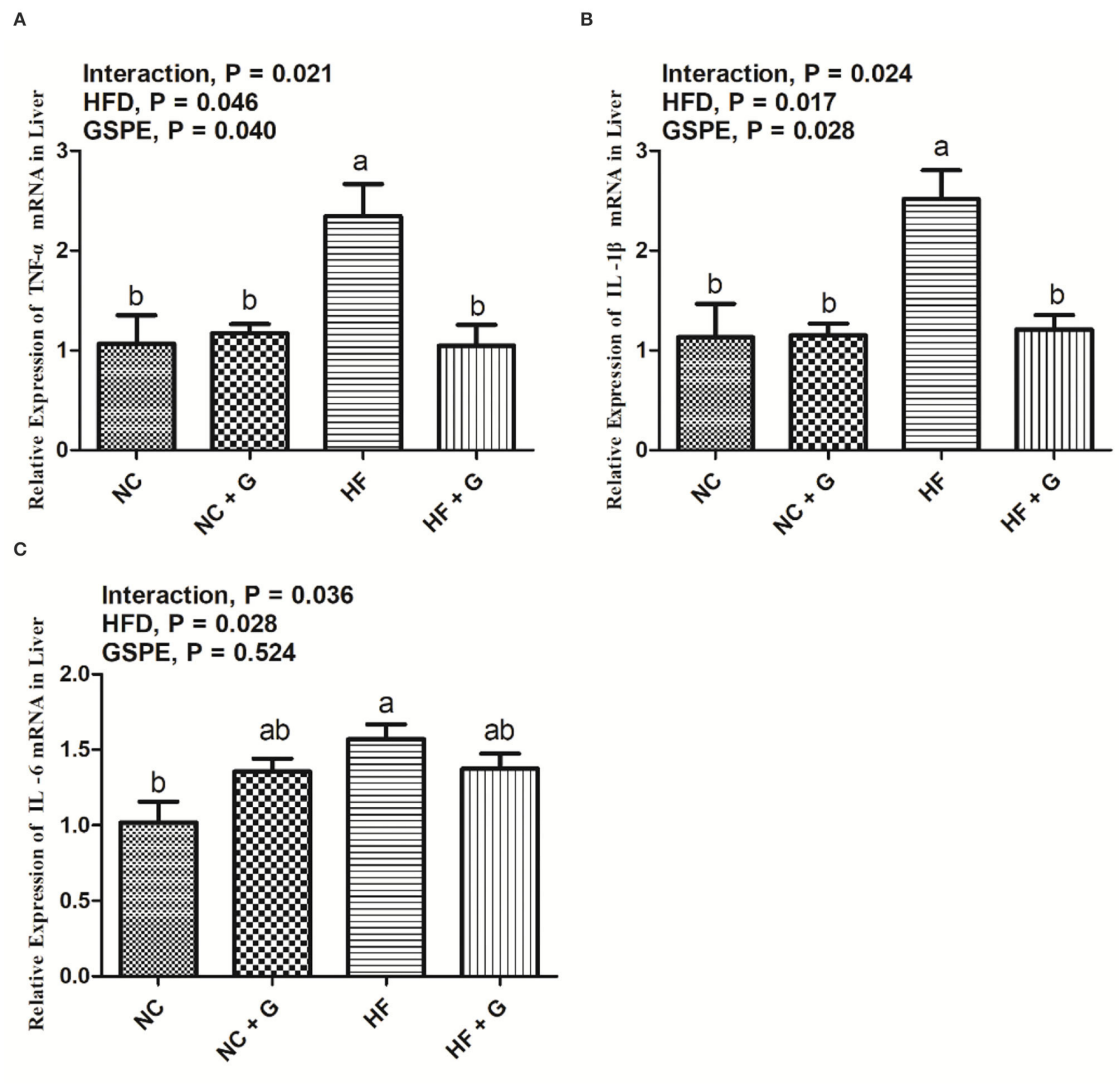
Effects of GSPE on relative expression of inflammatory cytokines of liver in rats fed long-term high-fat diet [**(A)** TNF-α mRNA expression; **(B)** IL-1β mRNA expression; **(C)** IL-6 mRNA expression]. Data were expressed as the mean ± SEM. Different letters means with different superscripts differ (*P* < 0.05). NC, standard diet; NC + G, standard diet + 500 mg/kg body weight GSPE; HF, high fat diet; HF + G, high fat diet + 500 mg/kg body weight GSPE.

The results of relative expression of inflammatory cytokines TNF-α, IL-1β, and IL-6 mRNA in rats' livers are shown in Figure 6. The HF diet significantly (*p* < 0.05) increased the expressions of TNF-α, IL-1β, and IL-6 mRNA than those in the basal diets. Also, GSPE supplementation reduced (*p* < 0.05) the TNF-α and IL-1β expressions but had no significant effects on IL-6 expression compared with these non-supplemented ones. Significant interactions were shown in all three parameters among the treatments. Similarly, mRNA expressions of TNF-α and IL-1β in the HF group had the highest (*p* < 0.05) level than the other three treatments. But the IL-6 mRNA expression in the HF diet only showed higher (*p* < 0.05) values than in the NC treatment.

### Effects of GSPE on the Relative Expression of MicroRNA-103 in the Liver of Rats Fed Long-Term High-Fat Diet

**Figure 7:**
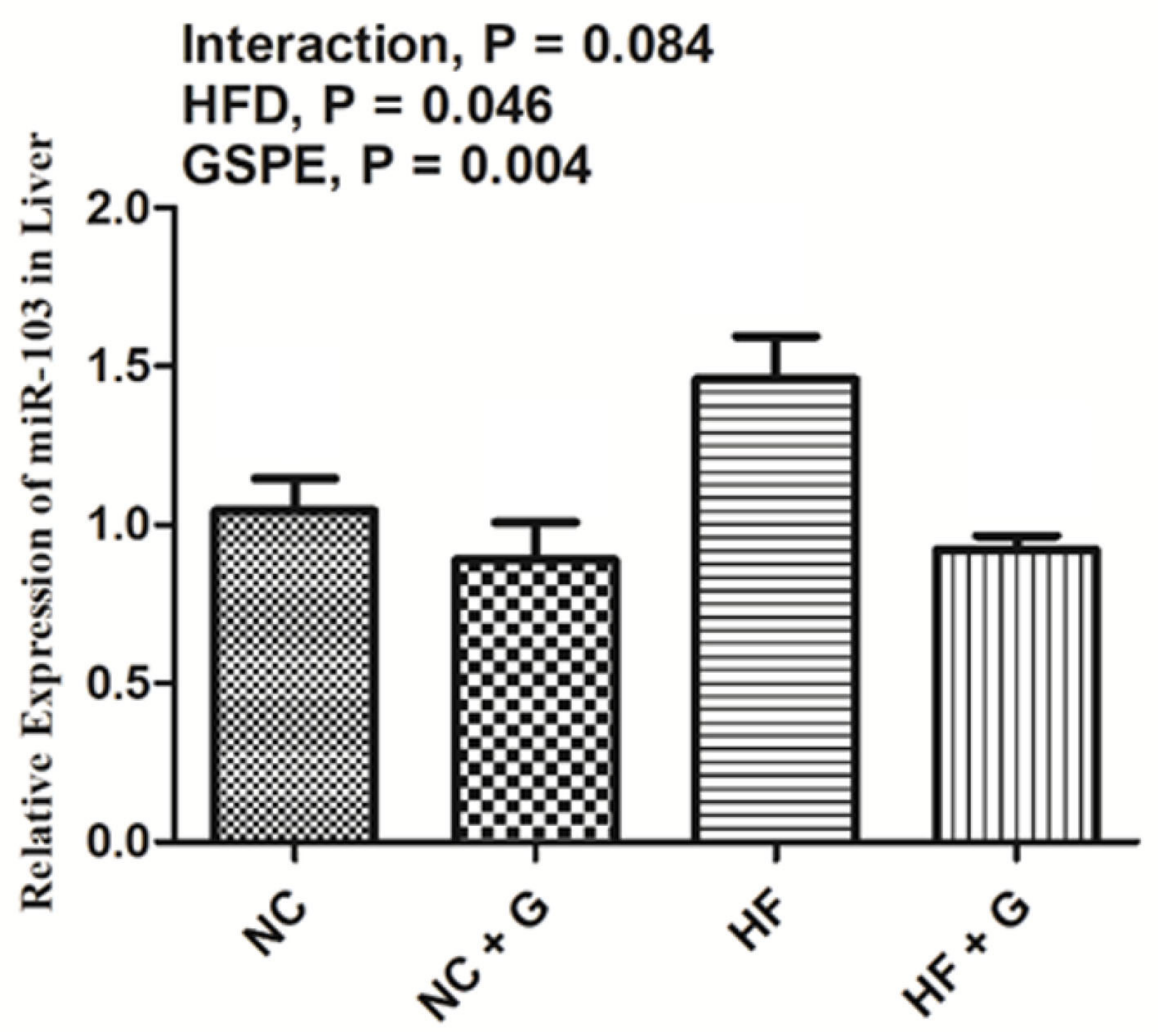
Effects of GSPE on relative expression of micro-RNA-103 of liver in rats fed long-term high-fat diet. Data were expressed as the mean ± SEM. Different letters means with different superscripts differ (*P* < 0.05). NC, standard diet; NC + G, standard diet + 500 mg/kg body weight GSPE; HF, high fat diet; HF + G, high fat diet + 500 mg/kg body weight GSPE.

The relative expression of miRNA-103 is described in Figure 7. Rats fed with HF diets observed higher (*p* < 0.05) expression of miRNA-103 than those fed with basal diets. Also, GSPE supplementation significantly (*p* < 0.05) decreased the expression compared with the non-supplemented ones. No interactions were observed in miRNA-103 expression in the liver.

### Effects of GSPE on Wnt3a/β-Catenin Pathway Proteins Concentration and Expression in the Liver of Rats Fed Long-Term High-Fat Diet

**Figure 8:**
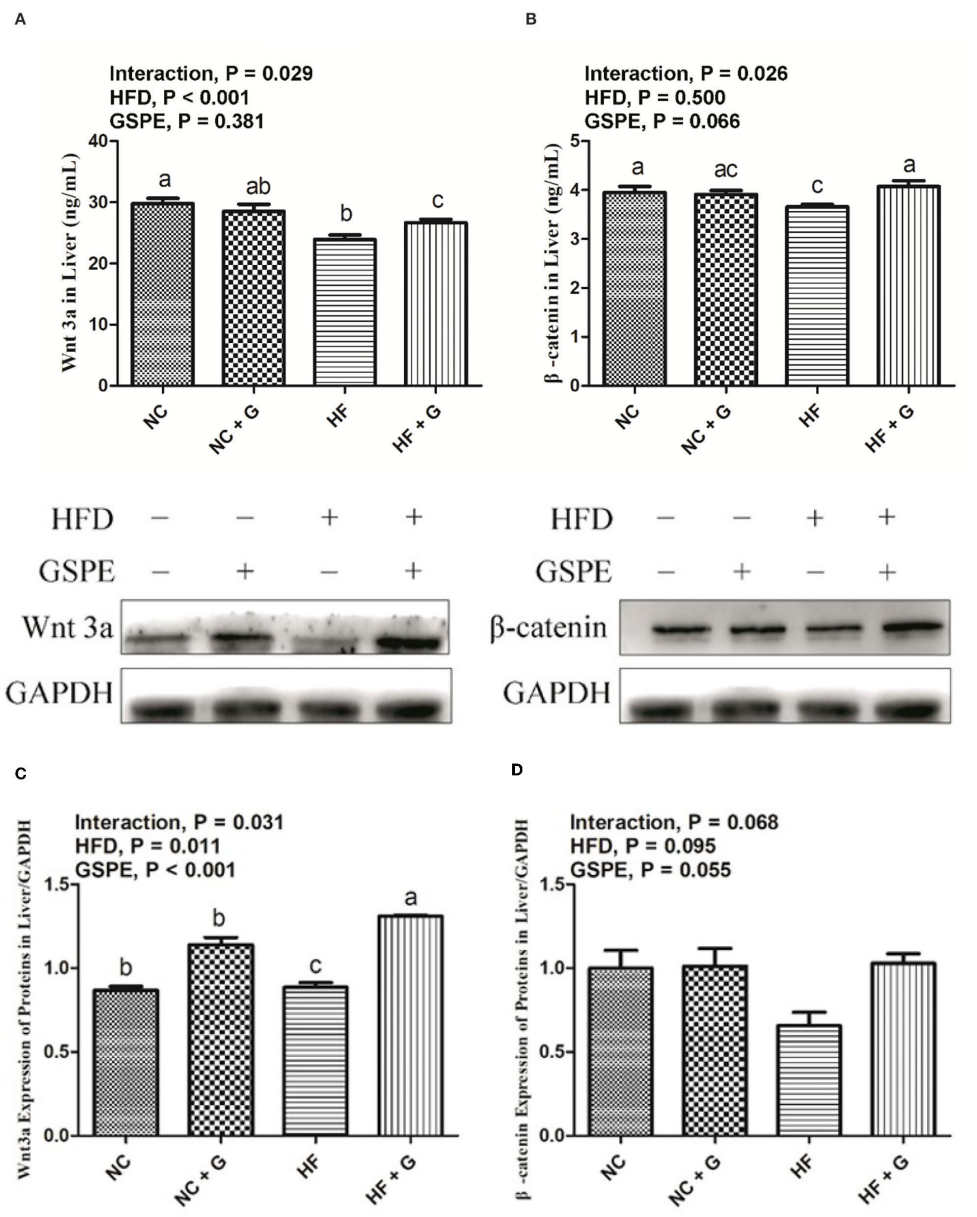
Effects of GSPE on Wnt3a/β-catenin pathway protein concentration and expression of liver in rats fed long-term high-fat diet [**(A)** Wnt3a protein concentration; **(B)** β-catenin protein concentration; **(C)** Protein production on Wnt3a; **(D)** Protein production on β-catenin]. Data were expressed as the mean ± SEM. Different letters means with different superscripts differ (*P* < 0.05). NC, standard diet; NC + G, standard diet + 500 mg/kg body weight GSPE; HF, high fat diet; HF + G, high fat diet + 500 mg/kg body weight GSPE.

The concentrations of Wnt3a/β-catenin pathway protein in rats' livers are shown in Figures 8A,B. There were no significant influences on GSPE supplementation among the treatments. Higher dietary fat significantly (*p* < 0.05) decreased the Wnt3a protein concentration (8A) in livers but did not affect the β-catenin protein concentration (8B). Significant interactions were observed in the Wnt3a and β-catenin protein concentrations between dietary fat level and GSPE. The HF group had the lowest protein concentrations on both Wnt3a and β-catenin compared with NC and HF + GSPE groups. However, the HF + GSPE group observed a lower (*p* < 0.05) concentration of Wnt3a protein than the NC group. As described in Figure 8C, higher dietary fat all resulted in higher (*p* < 0.05) expression of Wnt3a protein than those in normal diets. Similar results were also observed (*p* < 0.05) in GSPE groups compared with those of non-supplemental ones. Also, interactions were observed in the Wnt3a protein expression between dietary fat level and GSPE. The HF treatment had the lowest (*p* < 0.05) expression among the four treatments and the HF + GSPE group received the highest expression (*p* < 0.05) of Wnt3a protein among the other three groups. However, there were no significant effects on the β-catenin protein expression among the experimental treatments (Figure 8D).

## Discussion

In mammals, the liver plays a very important role in lipid metabolism. Pathologically, NAFLD mainly causes excessive deposition of liver fat and hepatic cell steatosis, and it was normally caused by a long-term high-fat diet (1, 18). Following the progression of NAFLD, metabolic problems including oxidative stress, inflammatory cytokine release, ERS, and apoptosis accompanied (2). GSPE is a natural polyphenolic compound primarily obtained from grape skin and seeds. It was indicated that GSPE plays an influential role in antioxidant, anti-inflammatory, and anti-apoptosis (19). The current experiment was designed to investigate the effects of GSPE on liver index and morphological, lipid metabolism, ERS, apoptosis, and Wnt3a/β-catenin pathway in rats fed with high-fat long-term diets, and subsequently provide a reference for the prevention and cure for NAFLD.

Obesity always leads to the adipose degeneration of the liver and it is also one of the main factors which cause NAFLD (20). In this experiment, rats in HF treatment had the highest liver indexes than other groups and their hepatocytes also showed adipose and injury such as circular lipid droplets, filled with fat, and inflammatory infiltration. These results demonstrated that these rats in the HF group had symptoms of NAFLD. At the same time, the addition of GSPE significantly decreased the liver index than those in HF treatment. This result was in accordance with the results from Khoshbaten et al. (8) who reported that oral GSPE reduced the liver volume and the steatosis level in people with NAFLD. Moreover, in the current study, it was observed that the supplementation of GSPE significantly relieved the steatosis of the liver and decreased the inflammatory infiltration. It provides proof of the liver protective responses triggered by the application of GSPE at the cellular level. In the liver, abnormal lipid deposition was attributed to the out-of-balance between lipid availability and consumption (21). Meanwhile, the metabolic disturbance of lipid and lipoproteins were also considered one of the etiological factors of NAFLD (22). PPARγ belongs to the nuclear receptor superfamily and plays an important role in adipogenesis (23). Horie et al. (24) reported that mice with liver-specific deficiency of PPARγ observed decreases in hepatic steatosis and lipogenic genes FAS. Similarly, the expression of PPARγ in the liver triggers the expression of adipocyte-specific genes and hepatic lipid accumulation (25). On the other hand, it was reported that the addition of GSPE could repress the expression of PPARγ. An *in vitro* experiment indicated that grape skin ethanolic extract treatment significantly reduced the expressions of the adipogenic genes including PPARγ in 3T3-L1 cells (26). A similar result was obtained in this experiment; GSPE addition decreased the PPARγ expression in the liver. It was known that PPARγ may induce the transcription of the fat synthesis gene FAS in steatosis livers (27). Previous studies suggested that GSPE also showed an ability to inhibit FAS activity by combination with β-ketoacyl reductase of FAS; whereas, in this study, no effects on FAS were observed (28). This may be due to the differences in concentrations and experiment models between studies.

MicroRNAs (miRNAs), are non-coding RNAs that are small and regulate gene expression at the post-transcriptional level. They are highly conserved between different species and binding to the 3' untranslated region of the target mRNA thereby controlling multiple signaling pathways at once (29, 30). It was suggested that miRNAs play important roles in the liver fatty acid homeostasis, adipogenesis, and lipid metabolism (31). In the current experiment, the HF diet improved the miR-103 expression and GSPE supplementation decreased the expression. The miR-103 was reported to have the ability to promote fat synthesis, and it was confirmed that it targeted regulating the mRNA expression of FAS (32, 33). However, Park et al. (34) reported that in diet-induced obese mice the expression of miR-103 was decreased. In line with this experiment, Joven et al. (7) indicated that the expression of miR-103 was increased in hyperlipidemic mice caused by diet. Moreover, they also observed that plant-derived polyphenols significantly reduced the miR-103 expression. Also, *in vitro* experiment conducted on the human hepatocellular carcinoma cell line Huh 7 reported that after apigenin treatment, the expression level of mature endogenous miR-103 decreased (35). It was suggested that the structure of polyphenols may determine their activity in fatty acid synthase (28). The inhibition of miR-103 expression in this experiment may be due to this, but further experiments are still needed to figure out the exact mechanisms of GSPE on the regulation of miRNAs.

The Wnt/β-catenin pathway is also known as the classical Wnt signaling pathway, which depends on the activation of β-catenin. It was suggested that this pathway plays extensive roles in animal biological processes including embryonic development, organization stability, metabolic balance, and cell maintenance (36). The inhibition of the Wnt pathway was considered to induce obesity and thereby cause metabolic disorder due to which it was reported to have the ability to inhibit adipocyte differentiation (37). In the current experiment, the results showed that in HF treatments the expressions of Wnt3a and β-catenin in the liver were significantly decreased which verified the function of the Wnt3a/β-catenin pathway in obesity. It was also observed that supplementation of GSPE improved the expression of Wnt3a and β-catenin protein in the liver. Owing to the lack of available studies, direct comparisons of response to GSPE on the Wnt3a/β-catenin pathway in obese rats are impossible. Recently, similar results were observed by Zang et al. (38) who reported that tea polyphenols activated the Wnt/β-catenin pathway in order to improve the liver fat deposition in obese adult zebrafish. Moreover, Zhang et al. (39) indicated that the miR-103 could target and modulate the Wnt3a by inhibiting the pathway of Wnt3a/β-catenin and reducing the lipidosis in preadipocytes. On the other hand, it was investigated that a large number of β-catenin expressed in the nucleus may cut the expression of adipogenic genes such as PPARγ thereby reducing the fat deposition (40). Therefore, the changes in expression of Wnt3a and β-catenin protein in the liver may be due to the feedback regulation of miR-103 and PPARγ in this experiment.

The activation of β-catenin also regulates the transcription of the apoptosis factors such as Bcl-2 and Bax (41). Also, along with the NAFLD development, hepatocyte apoptosis occurs. Apoptosis is one of the important steps in hepatic injury induced by NAFLD. It has been suggested that the aggravation of NAFLD *via* a complicated process had a high correlation with apoptosis, hepatic injury, fibrosis, and inflammation (42). The injury of the liver may cause excessive apoptosis and necrosis thereby triggering the inflammation and finally leading to fibrosis of the hepatocyte, eventually making the NAFLD worse. Therefore, the apoptosis and inflammation gene factors were also determined in the current experiment. The results show that the supplementation of GSPE reduced the mRNA expressions of Bax and proinflammatory factors TNF-α and IL-1β as well as improved the anti-apoptotic factor of Bcl-2 expression. Bcl-2 and Bax belong to the family of B-cell lymphoma-2. It has been suggested that they are the homologous, antagonist, and are the downstream target protein controlled by the Wnt/β-catenin signaling pathway (43). Some studies have observed that the activation of the Wnt/β-catenin pathway may inhibit apoptosis and hence protect the cells and organs (44, 45). For instance, Huang et al. (44) reported that in an alcoholic liver disease rat model, Wnt agonists stimulated the –catenin transduction and downregulated the expression of pro-apoptotic gene thereby protecting the liver from alcohol-induced apoptosis and damage. On the other hand, macrophage infiltration of these fat tissues such as M1 macrophages will secrete the pro-inflammatory factors including IL-6, TNF-α, and IL-1β aggravating the inflammatory response (46). The Wnt/β-catenin signaling pathway was also considered to have an anti-inflammatory function (47). Hatting et al. (48) suggested that the Wnt/β-catenin pathway played an important part that apoptotic gene knockout mice observed lower inflammatory. Similarly, Ma et al. (49) indicated that the Wnt/β-catenin pathway inhibited the expression of the proinflammatory factor IL-1β. Taken together, the results of this study substantiated the positive contribution of GSPE *via* the Wnt/β-catenin signaling pathway on liver cell apoptosis and inflammation.

Obesity leads to long-term low-intensity inflammation which results in continuous stress on the endoplasmic reticulum in cells (46). Meanwhile, hyper deposition of fat in cells set off the inhibition of protein which also causes the ERS (50). Recent research found that ERS plays a momentous role in NAFLD occurrence and development due to the accommodation of ERS on hepatocyte apoptosis (51). Chen et al. (52) observed that the ATF6 signaling pathway is one of the keys which affects the progression of NAFLD *via* regulating the ERS-induced inflammation and apoptosis. In the current experiment, the supplementation of GSPE significantly restrained the expressions of ATF6 and CHOP in the liver which was similar to previous studies. Gao et al. (53) reported that GSPE protected the nephrotoxicity by cisplatin through inhibition of the ERS-induced apoptosis. Recently, Long et al. (54) indicated that proanthocyanidins have positive effects in the small intestine of mice that protected the epithelial cells from zearalenone-induced apoptosis by ERS. Moreover, an *in vitro* study observed that the inhibition of the Wnt3a/β-catenin pathway enhanced the ERS and thereby improved the apoptosis of preadipocytes. Therefore, based on the results of this experiment, it was speculated that the suppression of ERS caused by hepatocyte apoptosis may be due to the positive expressions of the Wnt3a/β-catenin pathway.

## Conclusions

Long-term high-fat feeding caused non-alcoholic fatty liver disease and severe liver problems including megalohepatia, steatosis, inflammation, and hepatocyte apoptosis in rats. The supplementation of grape seed proanthocyanidin alleviated these symptoms. The results of the current experiment sustained that grape seed proanthocyanidin addition up-regulated the expression of the Wnt3a/β-catenin signaling pathway, thereby restraining the liver cell endoplasmic reticulum stress and hepatocyte apoptosis. It also promoted liver lipid metabolism including downregulating the expression of genes of lipid synthesis and inhibiting the liver fat deposition in rats. Furthermore, microRNA-103 may play a role in this signal-regulated pathway and this may be a worthy point for further research. In summary, liver protective effects were observed in grape seed proanthocyanidin and the current experiment provides a reference for the application of grape seed proanthocyanidin as a natural feed additive.

## Data Availability Statement

The original contributions presented in the study are included in the article/supplementary materials, further inquiries can be directed to the corresponding authors.

## Ethics Statement

The animal study was reviewed and approved by Animal Care and Use Committee of Northeast Agricultural University, People's Republic of China.

## Author Contributions

All authors listed have made a substantial, direct, and intellectual contribution to the work and approved it for publication.

## Funding

This research was supported by the National Key R&D Program of China (2018YFD0501204).

## Conflict of Interest

The authors declare that the research was conducted in the absence of any commercial or financial relationships that could be construed as a potential conflict of interest.

## Publisher's Note

All claims expressed in this article are solely those of the authors and do not necessarily represent those of their affiliated organizations, or those of the publisher, the editors and the reviewers. Any product that may be evaluated in this article, or claim that may be made by its manufacturer, is not guaranteed or endorsed by the publisher.

## Abbreviations

NAFLD: non-alcoholic fatty liver disease
GSPE: grape seed proanthocyanidin
ERS: endoplasmic reticulum stress
NC: standard diet
HF: high-fat diet
PPARγ: peroxisome proliferators activate the receptor γ
FAS: fatty acid synthetase
ATF6: activating transcription factor 6
CHOP: CCAAT/enhancer-binding protein homologous protein
TNF-α: tumor necrosis factor α
IL-1β: interleukin-1β
IL-6: interleukin-6.
